# Investigating the efficacy of an online mindfulness-based intervention in a sample of medical students with obsessive-compulsive disorder

**DOI:** 10.1192/j.eurpsy.2025.1719

**Published:** 2025-08-26

**Authors:** J. Rodrigo, A. Hassoulas, E. Forty, P. Palmer

**Affiliations:** 1 Cardiff University, Cardiff, United Kingdom; 2 School of Medicine; 3 Institute of Psychological Medicine and Clinical Neurosciences, Cardiff University, Cardiff, United Kingdom

## Abstract

**Introduction:**

Obsessive-compulsive disorder (OCD) affects 1-3% of the population and is the fourth most debilitating psychiatric disorder. OCD characterised by persistent obsessions and compulsions in the ICD-11 is more common in students, with rates often exceeding 3-4% in medical students. Effective mental health services and interventions are critical in supporting these students.

**Objectives:**

Creating an online mindfulness-based intervention for Cardiff University medical students to engage with remotely. Evaluating the effectiveness of the intervention in reducing OCD symptoms.

**Methods:**

Medical students at Cardiff University’s School of Medicine, including those in intercalation years, were invited to fill out an online survey using Microsoft Forms. This included self-report measures such as the Beck Depression Inventory-II (BDI-II), the State-Trait Anxiety Inventory (STAI), and the Obsessive-Compulsive Inventory-Revised (OCI-R). Recruited participants completed a two-part intervention based on Acceptance and Commitment Therapy (ACT). The course was designed on the Xerte platform and used reflective tasks, interactive elements, and embedded videos. The intervention was developed with MyMedic, the Medical School’s mental health service. Participants completed the same online survey and a feedback form post intervention. Responses were analysed for changes in OCI-R scores.

**Results:**

Thirty-two students completed the pre-intervention survey. Six students met the study’s inclusion criteria. A significant positive relationship was found between OCI-R scores (minus hoarding) and BDI-II scores (χ² (3, N=32) = 10.745, p=0.01) (Figure 1). Three participants revealed reduced OCI-R (minus hoarding), STAI, and BDI-II scores after the intervention (Figure 2). The intervention was rated highly for usefulness and relevance, but neutral for interactivity. The embedded videos were deemed useful, and the module was thought-provoking.

**Image 1:**

**Figure:**
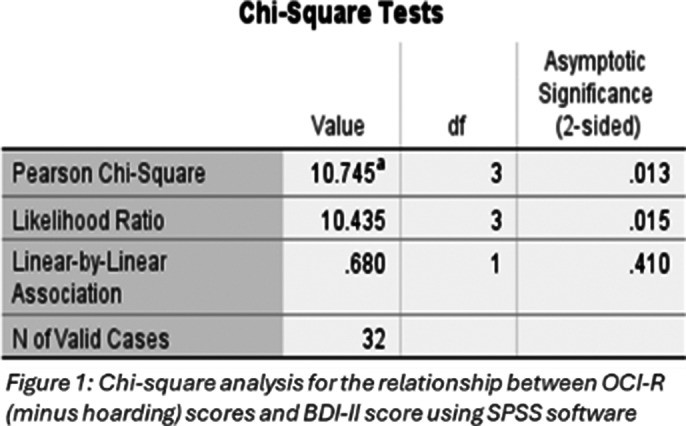

**Image 2:**

**Figure:**
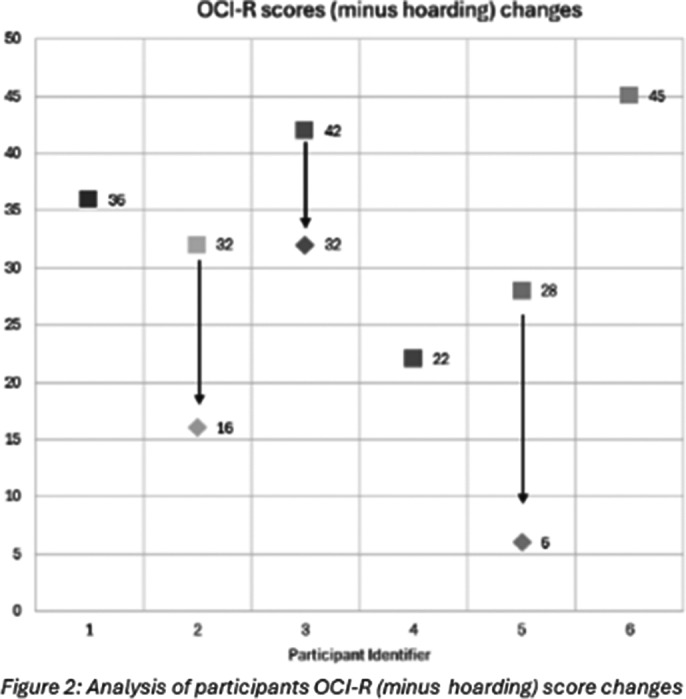

**Conclusions:**

This study found an 18% prevalence of OCD among medical students. The ACT-based skills course which emphasises psychological flexibility and mindfulness resulted in a significant reduction in the OCI-R. ACT could be a useful tool for university support services, potentially complementing or replacing CBT. Integrating such interventions into medical curricula may provide more comprehensive support and reduce wait times for mental health services.

**Disclosure of Interest:**

None Declared

